# Supplementary material for: Self-Reported Anxiety and Depression among Parents of Primary School Children during the COVID-19 Pandemic in Thailand, 2022
Source: Int J Environ Res Public Health. 2023 Apr 24;20(9):5622. doi: 10.3390/ijerph20095622 (PMC10178323; doi:10.3390/ijerph20095622)
Supplement: Supplementary file 1 [file ijerph-20-05622-s001.zip › ijerph-2274225-SI.pdf]

Supplementary file S1: Participants by school type, location, and questionnaire types

Table S1a: Percentages of participants by school type and location

Province	School		N	%
	Public	Private		
Bangkok	12	43	55	7.8
Chiang Rai	50	45	95	13.5
Udon Thani	229	138	367	52.4
Chonburi	48	43	91	13.0
Songkhla	50	43	93	13.3
**Total**	**389**	**312**	**701**	**100.0**

Table S1b: Percentages of participants in the survey by school location and type of questionnaires

Province	Type of questionaries				Total
	paper-based (n)	%	online (n)	%	
Bangkok	43	78.2	12	21.8	55
Chiang Rai	95	100.0	0	0.0	95
Udon Thani	0	0.0	367	100.0	367
Chonburi	0	0.0	91	100.0	91
Songkhla	93	100.0	0	0.0	93
**Total**	**231**	**32.9**	**470**	**67.1**	**701**
